# Promoting the use of self-management in patients with spine pain managed by chiropractors and chiropractic interns: barriers and design of a theory-based knowledge translation intervention

**DOI:** 10.1186/s12998-019-0267-6

**Published:** 2019-10-16

**Authors:** Owis Eilayyan, Aliki Thomas, Marie-Christine Hallé, Sara Ahmed, Anthony C. Tibbles, Craig Jacobs, Silvano Mior, Connie Davis, Roni Evans, Michael J. Schneider, Heather Owens, Fadi Al Zoubi, Jan Barnsley, Cynthia R. Long, Andre Bussières

**Affiliations:** 1 School of Physical and Occupational Therapy, McGill University, 3654 Promenade Sir-William-Osler, Montreal, Quebec H3G 1Y5 Canada; 2 Centre for Interdisciplinary Research in Rehabilitation of Greater Montreal (CRIR), 6363, Hudson Road, office 061, Lindsay Pavilion of the IURDPM, Montreal, QC H3S 1M9 Canada; 3 Canadian Memorial Chiropractic College, 6100 Leslie St, North York, ON M2H 3J1 Canada; 4 University of British Columbia, 2329 West Mall, Vancouver, BC V6T 1Z4 Canada; 5 Centre for Collaboration, Motivation and Innovation, PO Box 1343, Vernon, BC V1T 6N6 Canada; 6 University of Minnesota, Minneapolis, MN 55455 USA; 7 University of Pittsburgh, 4200 Fifth Ave, Pittsburgh, PA 15260 USA; 8 University of Toronto, 27 King’s College Cir, Toronto, ON M5S Canada; 9 Palmer College, Davenport, 1000 Brady St, Davenport, IA 52803 USA

**Keywords:** Spine pain, Chiropractic, Self-management, Theory-based intervention, Knowledge translation, Theoretical domains framework

## Abstract

**Background:**

The literature supports the effectiveness of self-management support (SMS) to improve health outcomes of patients with chronic spine pain. However, patient engagement in SMS programs is suboptimal. The objectives of this study were to: 1) assess participation in self-care (i.e. activation) among patients with spine pain, 2) identify patients’ barriers and enablers to using SMS, and 3) map behaviour change techniques (BCTs) to key barriers to inform the design of a knowledge translation (KT) intervention aimed to increase the use of SMS.

**Methods:**

In summer 2016, we invited 250 patients with spine pain seeking care at the Canadian Memorial Chiropractic College in Ontario, Canada to complete the Patient Activation Measure (PAM) survey to assess the level of participation in self-care. We subsequently conducted individual interviews, in summer 2017, based on the Theoretical Domains Framework (TDF) in a subset of patients to identify potential challenges to using SMS. The interview guide included 20 open-ended questions and accompanying probes. Findings were deductively analysed guided by the TDF. A panel of 7 experts mapped key barriers to BCTs, designed a KT intervention, and selected the modes of delivery.

**Results:**

Two hundred and twenty-three patients completed the PAM. Approximately 24% of respondents were not actively involved in their care. Interview findings from 13 spine pain patients suggested that the potential barriers to using SMS corresponded to four TDF domains: *Environmental Context and Resources; Emotion; Memory, Attention & Decision-Making; and Behavioural Regulation*. The proposed theory-based KT intervention includes paper-based educational materials, webinars and videos, summarising and demonstrating the therapeutic recommendations including exercises and other lifestyle changes. In addition, the KT intervention includes Brief Action Planning, a SMS strategy based on motivational interviewing, along with a SMART plan and reminders.

**Conclusions:**

Almost one quarter of study participants were not actively engaged in their spine care. Key barriers likely to influence uptake of SMS among patients were identified and used to inform the design of a theory-based KT intervention to increase their participation level. The proposed multi-component KT intervention may be an effective strategy to optimize the quality of spine pain care and improve patients’ health-outcomes*.*

**Electronic supplementary material:**

The online version of this article (10.1186/s12998-019-0267-6) contains supplementary material, which is available to authorized users.

## Background

Spine pain is a leading cause of disability and work absenteeism worldwide [1–4]. Between 50 and 80% of adults suffer from spine pain at least once during their lives [5, 6]. Spine pain impacts both society and individuals with physical, psychological, and emotional burden [5, 7–15]. Between 1990 and 2015 the number of “years lived with disability caused by low back pain increased by 54%” [1]. The direct cost of spine pain is approximated at $6 to $12 billion annually in Canada [16].

Spine pain is often a chronic condition, and self-management support (SMS) could reduce its consequences on patients’ health [17]. Self-management is defined as an “individual’s ability to manage the symptoms, treatment, physical and psychosocial consequences, and lifestyle changes inherent in living with a chronic condition” [18]. SMS strategies aimed at helping patients develop the skills they need to change negative thinking and increase physical activity [19, 20] are the keys to effectively manage spinal pain [21–28]. Clinical practice guidelines (CPGs) recommend SMS for patients with spine pain [29–32]. Current evidence suggests SMS has similar treatment effects compared to other more expensive and intensive approaches such as massage, acupuncture, yoga, and exercise [17, 33]. SMS helps reduce pain, disability, and psychological distress [17, 34].

Many patients with spine pain consult primary care providers including chiropractors for relief [35–37] of back and neck pain. Nearly half of these patients (median of 49.7% (interquartile range (IQR): 43.0–60.2%)) consult specifically for low back pain or back problems, and another quarter (22.5%, IQR, 16.3–24.5%) for neck problems [37]. Among those patients seeking chiropractic care for complaints of spine pain in Canada, some consult chiropractic interns and supervisory clinicians in outpatient teaching clinics [38].

Globally, the routine use of evidence-based recommendations, including the delivery of SMS, remains suboptimal among practicing chiropractors [39–42]. Barriers among chiropractic clinicians and interns to using SMS in a large chiropractic teaching institution in Canada were previously assessed in a related qualitative study [42]. Key barriers corresponding to the theoretical domain framework (TDF) included: *Knowledge; Skills; Environmental context and resources; Emotion; Beliefs about Capabilities; Memory, attention & decision making;* and *Social Influence*. Patients’ compliance with SMS recommendations is also suboptimal [34, 43, 44], and there is a shortage of studies that assess the barriers to the use of SMS among individuals with spine pain. Nonetheless, known barriers to implementing SMS among patients with chronic pain, low back pain, or osteoarthritis include: low self-efficacy, negative beliefs and lack of readiness to using SMS, lack of time, family commitments, poor emotional status, and poor access to information on SMS [45–47]. Poor communication between clinicians and patients also limits the use of SMS [47]. Collectively, these barriers can decrease both the utilisation of - and adherence to - SMS, and ultimately reduce its effectiveness.

Successful implementation of SMS approaches requires patients to change unhealthy behaviours and commit to healthier ones [19, 20], but changing individuals’ behaviour is challenging [40, 48]. Therefore, it is important to assess patients’ knowledge of SMS, skills, and confidence in managing their own health; a phenomenon referred to as activation level in self-care [49]. Information on patient activation level can inform the design of appropriate strategies to support behaviour change [49]. In addition, understanding the barriers that patients face when using SMS in chiropractic contexts is necessary for successful implementation of SMS programs through theory-based Knowledge Translation (KT) interventions. The literature suggests that theory-based KT interventions can increase patients’ compliance with recommended care [50], increase the likelihood of successful behaviour change [51–54], and lead to better health outcomes in different healthcare settings [55]. To date, few studies have assessed the barriers to using SMS among patients with spine pain in chiropractic teaching institutions.

The objectives of this study were to: 1) assess participation in self-care (i.e. activation) among patients with spine pain, 2) identify patients’ barriers and enablers to using SMS, and 3) map behaviour change techniques (BCTs) to key barriers to inform the design of a KT intervention aimed at increasing the use of SMS.

### Conceptual framework

The Theoretical Domains Framework (TDF) was originally developed to assess the factors likely to influence professional behaviour change. The TDF has been used extensively across a range of patient populations, settings, and health conditions to understand implementation challenges [56–58], and to guide the development of theory-based KT interventions [55, 59–62]. The TDF integrates 33 theories of behaviour change and identifies 14 factors most likely to affect behaviour change: *Knowledge, Skills, Social/Professional Role and Identity, Beliefs about Capabilities, Optimism, Beliefs about Consequences, Reinforcement, Intentions, Goals, Memory/Attention and Decision Processes, Environmental Context and Resources, Social Influences, Emotion,* and *Behavioural Regulation* [63].

## Methods

### Study design

Three-phase mixed-methods sequential transformative design comprised of quantitative and qualitative data collection, and analyses. Ethical approval was obtained from the Research Ethics Board of both McGill University (McGill IRB: A08-E54-16B) and the Canadian Memorial Chiropractic College (CMCC-REB Approval 1512B02). Written informed consent was obtained from all participants.

### Setting

The study took place at the Canadian Memorial Chiropractic College (CMCC) campus clinic and two CMCC-affiliated external teaching clinics (CMCC’s Clinic at Sherbourne Health and South Riverdale Community Health Centre) in Toronto, Ontario. CMCC is the largest chiropractic teaching institution in Canada. Three other CMCC-intercity clinics were excluded because they were either not engaged in research activities or they declined to participate.

### Approach

A systematic approach proposed by French et al. [55] was used to guide the development of a theory-based intervention aimed at optimizing the use of SMS among patients with spine pain. The approach includes 4 questions:

1. Who needs to do what, differently? For this question, current literature suggests that the use of SMS among patients is suboptimal [45–47]. Such a evidence-practice gap was confirmed by research team members overseeing CMCC clinical activities [64]. This question was further addressed in ***phase 1*** of the current study which aimed to assess patient activation in SMS (Quantitive approach).
2. Using a theoretical framework (i.e. TDF [63]), which barriers and enablers need to be addressed?
3. Which intervention components (behaviour change techniques and mode(s) of delivery) could overcome the modifiable barriers and enhance the enablers?
4. How can behaviour change be measured and understood? This question is beyond the scope of this paper and thus not addressed.

Questions 2 and 3 were addressed in two distinct phases: ***phase 2*** aimed to identify barriers and enablers to the use of SMS (Qualitative approach). The interview data was used to inform the design of a theory-based KT intervention (phase 3). In ***phase 3***, we mapped BCTs to relevant barriers and enablers identified in phase 2.

## Phase 1: estimating patient activation (quantitative data)

### Participants

During the summer of 2016 we assembled a convenience sample of consecutive patients seeking care at four CMCC outpatient clinics was assembled for Phase 1. Patients had to meet the following inclusion criteria: ages of 18–70 years, currently receiving treatment from a consenting chiropractic intern, having received at least three treatment sessions for a primary back or neck pain complaint, and be able to read and hold a conversation in English.

### Data collection

#### Study instrument

The Patient Activation Measure (PAM), a self-administered 13 item questionnaire, was used to assess the level of activation in self-care among patients with spine pain [65]. The PAM divides patients into one of four activation levels (Table 1), which are associated with specific self-management and other health related behaviours [67]. The PAM is used to indicate the level of patient participation in SMS [65], it has been extensively tested among individuals with different health conditions, and is a reliable and valid tool [66, 68, 69]. We also asked eight additional “short answer” questions: age, gender, duration of spine pain, previous chiropractic care, SMS knowledge, Brief Action Planning (BAP) knowledge, and any existing medical comorbidities. The multiple regression analyses showed that having knowledge of SMS and knowledge of BAP were significantly associated with a higher score on the PAM. More information on the regression analysis can be found in Additional file 1.

**Table 1:** “The four levels of patient activation” [66]

Level	Description
Level 1	Individuals tend to be passive and feel overwhelmed by managing their own health. They may not understand their role in the care process.
Level 2	Individuals may lack the knowledge and confidence to manage their health
Level 3	Individuals appear to be taking action but may still lack the confidence and skill to support their behaviours.
Level 4	Individuals have adopted many of the behaviours needed to support their health but may not be able to maintain them in the face of life stressors

### Procedures

Eligible patients with spine pain were identified using OSCAR [70], an electronic medical records system. Chiropractic interns were asked to recruit consecutive patients who were seeking care for spine pain. The CMCC research coordinator contacted 250 potentially interested patients. Eligible patients (*n* = 223) were verbally informed of the study aims and consented appropriately. The patients were also asked to read the online written consent form prior to completing the PAM survey questions using an iPad and computers at the clinics. Patients agreed to participate by clicking “yes” on the screen.

### Sample size and data analysis

Descriptive statistics were used to obtain the PAM average total score and distribution using SAS version 9.4 (North Carolina University, USA) [71]. The total score (calibrated score 0 (no activation) - 100 (high activation)), was calculated by the Insignia Health Group (licensee of the PAM questionnaire) [66].

Sample size needed to assess the patients’ participation in SMS follows the equation of N ≥ (1.96)^2^ * SD^2^ / d^2^ [72]. The SD was determined based on the PAM results of previous studies [73, 74]. The required sample size was a total of 216 subjects; (1.96^2^ * 15^2^) / 2^2^.

## Phase 2: identifying barriers and enablers (qualitative data)

Subsequent to Phase 1, we conducted semi-structured individual interviews with spine pain patients in the summer of 2017. Interviewees were selected from a purposive sample of phase 1 participants. The interview guide was designed using the TDF framework [63] (See Additional file 2).

### Participants

A purposive sample of patients with spine pain was assembled from two CMCC clinics, which had a larger number of patients and clinicians who were more familiar with the recruitment process. The sociodemographic characteristics of patients were similar across participating clinics. To ensure variety amongst respondents, the invitation email was sent to different groups of patients using the following criteria: age, gender and duration of spine pain.

### Data collection

#### Interview guide

The interview guide was developed based on the TDF [63] to understand the patients’ involvement in SMS (described in phase 1) and to identify their perceived barriers and enablers to the use of SMS. Specifically, the interview guide aimed to explore: patients’ beliefs about spine pain and recovery, SMS information/tools needed and factors influencing adherence to SMS, activity limitation, exercise and physical activity, and verbal communication with their care providers. The interview guide included 20 open-ended questions, 1–2 questions per domain with accompanying probes, informed by a previous study on a related topic [75]. These questions covered 13 of the 14 TDF domains; the *“Social/Professional Role and Identity”* domain was excluded as it relates only to clinicians. Five KT experts and clinicians assessed the content validity of the interview guide. Each interview took approximately 30 min.

#### Procedures

Thirty-eight patients attending CMCC outpatient clinics were deemed eligible to participate in the interview as they met the inclusion criteria listed under phase 1. A research assistant then contacted a purposive sample of 19 patients by email inviting them to participate in an individual interview. Online interviews were conducted with the first 13 patients who responded favourably to the invitation email. Thirteen participants is considered an appropriate sample size for achieving saturation in a theory-based interview study [76]. To assess if we achieved data saturation, we analysed the interview data for the 13 consecutive patients. No new themes emerged after the 11th interview, which confirmed that thematic saturation had been achieved.

The interviews were conducted online using Cisco WebEx™ (Milpitas, California (United States)) by the research assistant (MSc degree) who had previous experience in facilitating interviews based on the TDF. All interviews were audio recorded, transcribed verbatim and anonymized. All participants completed and signed an informed consent form prior to the interview.

### Data analysis

Two assessors independently coded each transcript deductively. The assessors met three times online to compare the results. Disagreements were formally resolved by a third team member (AB). The analysis was similar to the one used by the research team in prior studies of chiropractors [62, 77]. Briefly, each transcript was divided into different statements (unit of meaning), after which each statement was coded into relevant TDF domains onto an Excel spreadsheet. Statements were then linked to specific beliefs. A specific belief is defined as “a core statement that captures a common theme from multiple response statements and provides detail about the role of a given domain in influencing practice behaviour” [62, 78]. The specific beliefs were classified into one of 3 categories: likelihood to either increase (facilitator), decrease (barrier), or have no influence on the use of SMS. The most important key **barriers** were identified as: considering frequency of belief, importance of the belief, and contrasting beliefs.

## Phase 3: intervention design

The aim of phase 3 was to design a KT intervention to address previously identified barriers from phase 2 using intervention mapping.

### Participants

Seven research team members attended a half-day online meeting. Members included three KT experts, one patient representative, and three CMCC faculty members: a health services researcher, the Dean of Clinics, and a person overseeing curricular development. All CMCC faculty members were familiar with the TDF and BCTs.

### Procedure

Guided by established methods for designing KT interventions based on TDF-informed problem analysis [56, 79, 80], three team members (AB, AT, OE) mapped the key TDF barriers identified in Phase 2 onto relevant BCTs. Intervention components corresponding to the selected BCTs that could potentially address the key barriers were considered and selected [42, 62]. The remaining team members were then provided with the overall findings (i.e. key barriers and mapping exercise) in advance of a group meeting and asked to review each step, before recommending other potentially effective KT intervention components. Based on the available evidence of the effectiveness of BCTs [81–84] and the ease of implementation of such interventions into clinical settings (e.g., extent to which the proposed intervention is deemed to be acceptable, appropriate, and feasible) [85], the group reached a consensus on the preferred KT intervention components and modes of delivery (e.g. paper-based/electronic format).

The resultant selected KT interventions aimed mainly to facilitate the implementation of SMS guided by the BAP technique [86]. The BAP was developed based on motivational interviewing [86], and has been used in different clinical settings and integrated into the curricula of several medical training programs to enable the utilisation of SMS [86, 87]. Furthermore, BAP could be an ideal SMS program for the busy clinician [86]. The BAP technique includes three questions and five skills including the “offering of a behavioral menu, SMART planning, eliciting a commitment statement, problem solving for low confidence, and follow up” [88]. The technique consists of engaging a dialogue between the patient and healthcare providers in order to help patients take care of their own health.

## Results

### Phase 1—PAM survey

Data were collected from 223 of 250 (89.2% response rate) spine pain patients. The majority of respondents were female (*n* = 125/219; 57.1%), with a mean age of 49.4 years (SD = 14.1). Nearly 80% (*n* = 175/219) reported having spine pain greater than 1 year in duration (Table 2). The average PAM score was 64.5 (SD =12.9), suggesting a good level of activation. While respectively 103/219 (47%) and 64/219 (29.2%) participants reported scores corresponding to the third and fourth levels (i.e., high activation level), approximately a quarter (*n* = 52/219, 23.7%) of participants were in the first or second levels (low activation level). The raw data are presented in Additional file 3.

**Table 2:** Survey Participants Characteristics (*n* = 219)

Variable	Mean (SD), N (%)
Age	49.4 (14.1)
Gender, women	125/219 (57.1%)
Duration of Spine Pain: < 1 year	44/219 (20.1%)
> 1 year	175/219 (79.9%)
Received Previous Chiropractic Care (yes)	169/219 (77.2%)
Medical conditions beside back/neck pain (yes)	138/218 (63.3%)
PAM score	64.5 (12.9)
PAM Activation Levels: Level 1	19/219 (8.7%)
Level 2	33/219 (15.1%)
Level 3	103/219 (47%)
Level 4	64/219 (29.2%)

*PAM* patient activation measure

### Phase 2—interviews

#### Characteristics of participants interviewed

The 13 interviewed participants had a mean age of 48 ± 15.6 years (range 23–67 years); 61% (8/13) were women, and their average duration of spine pain was 13.8 ± 14.7 years.

#### Key themes identified within relevant domains

We identified 457 statements representing 44 specific beliefs and 20 themes (see Additional file 4). Additional file 5 provides interviewees’ quotes categorized by specific domains and beliefs. Five key TDF domains were presumed to have an important influence on the targeted behaviour: 1) Environmental Context and Resources; 2) Emotion; 3) Memory, Attention & Decision Making; 4) Behavioural Regulation; and 5) Knowledge.

### Phase 3—intervention design

Different BCTs were mapped to key relevant domains, and KT intervention components and actions were proposed based on those BCTs (Table 3). Based on effectiveness and feasibility considerations, team members agreed on the modes of delivery for the KT intervention.

**Table 3:** Mapping behaviour change techniques on key domains, proposed KT interventions and actions

Self-management-TDF barriers (BCTs)	KT Intervention	Actions
Knowledge - Patients believe that they know enough about SMS *Behavioural change techniques:* *Information regarding behaviour, outcome.*	- Standardized materials as tools to introduce the concept of SMS	- Context: the majority of patients believe that exercise is the core component of self-management strategies (SMS) - Aim: to introduce and maximize the patients’ knowledge on SMS - Opportunities: • Distribute educational materials to patients summarizing the components of SMS, and emphasizing ways to initiate/sustain life style changes • Webinar to provide information on SMS • Provide information on SMS on social media (e.g. Facebook)
Environmental Context and Resources - Lack of time to use self-management among patients - Not receiving educational materials *Behavioural change techniques:* *(Environmental Changes; Time management)*	- Standardized electronic or printed materials as tools for facilitating the use of SMS - Persuasive communication	- Context: Lack of time is a barrier to using SMS - Aim: helping patients better manage their time to use SMS - Opportunity: • Encouraging patients to seek advice from clinicians/interns on ways to manage their time to be able to use SMS (before going to work/school, at lunch time, going to the gym …) • Provide patients with educational materials (paper based and e-pamphlets) summarizing the key components of self-management • Webinar and videos to provide information on SMS
Emotion - Feeling of anxiety/ frustrating regarding use SMS *Behavioural change techniques:* *(Stress Management, Social support (emotional); Coping strategies)*	- Modeling, demonstration of behaviour by others	- Context: patients stated that they have some anxiety and concern regarding performing therapeutic exercises improperly - Aim: minimizing the anxiety among patients by providing them with materials summarizing the prescribed exercises - Opportunities: • Webinar and videos to provide information about the SMS and to demonstrate home exercises • Having them speak to other patients who have been successful ✓ create a patient video to share stories on successful strategies on how to integrate SMS into daily schedule
Memory, attention & decision making - Few patients were not involved in the decision making - Remembering to do SMS is challenging *Behavioural change techniques:* *planning/implementation; Prompts/Triggers/Cues; Motivational Interviewing*	- Persuasive communication and information regarding behavioural outcomes - Provision of information - Instructions - Reminders	- Context: Some patients were not involved in the decision making process. Many patients have difficulty in remembering the SMS components - Aim: to facilitate the involvement of patients in the decision making process, and to remind patients about the use of SMS - Opportunities: • Distribute educational/instructional materials to patients to facilitate the shared decision making • Encourage patients to be actively involved in the decision making process by using the principles of BAP • Webinar to provide suggestions on ways of implementing SMS • Social media to provide information on SMS • Send patients reminders to use SMS via: ▪ E-mail ▪ Phone call (ex. when confirming next appointment)
Behavioural Regulation - Some patients don’t use SMS as much as they should *Behavioural change techniques:* *planning/implementation; Prompts/Triggers/Cues*	- Reminders - Persuasive communication	- Context: Some patients do not use SMS as often as they should - Aim: to understand why they don’t and motivate patients to use SMS more regularly - Opportunities: • Encourage clinicians/interns to help patients form a SMART plan to self-manage • Encourage patients to put Post-it notes near their computer, automated recalls on their cell phone and/or lap-top • Send patients reminders to use SMS via: ▪ E-mail ▪ Phone call

*BAP* brief action planning, *BCT* behavioural change technique, *KT* knowledge translation, *SMS* self-management strategy

#### Key TDF domains (phase 2) and proposed KT intervention components (phase 3)

In this section, we first present the *key TDF domains* with the corresponding specific beliefs and themes (*Phase 2*). Next, we mapped the BCTs onto these key domains and the corresponding intervention components proposed by the team members (*Phase 3*). Table 4 summarizes the key TDF domains and proposed KT intervention.

**Table 4:** The key TDF domains and proposed KT intervention

Self-management-TDF barriers	Description	Proposed KT intervention
Knowledge - Patients believe that they know enough about SMS	- Context: the majority of patients believe that exercise is the core component of self-management strategies (SMS)	• Distribute educational materials to patients summarizing the components of SMS, and emphasizing ways to initiate/sustain life style changes • Webinar to provide information on SMS • Provide information on SMS on social media (e.g. Facebook)
Environmental Context and Resources - Lack of time to use self-management among patients - Not receiving educational materials	- Lack of time was a barrier to using SMS	• Encourage patients to seek advice from clinicians/interns on ways to manage their time to be able to use SMS (before going to work/school, at lunch time, going to the gym …) • Provide patients with educational materials (paper based and e-pamphlets) summarizing the key components of self-management • Webinar and videos to provide information on SMS
Emotion - Feeling of anxiety/ frustration regarding use SMS	- Patients stated that they have some anxiety and concern regarding performing therapeutic exercises adequately	• Webinar and videos to provide information about the SMS and to demonstrate home exercises • Have patients speak to other patients who have been successful ✓ create a patient video to share stories on successful strategies on how to integrate SMS into daily schedule
Memory, attention & decision making - Few patients were not involved in the decision making - Remembering to do SMS is challenging	- Some patients were not involved in the decision making process. Many patients had difficulty in remembering the SMS components	• Distribute educational/instructional materials to patients to facilitate the shared decision making • Encourage patients to be actively involved in the decision making process by using the principles of BAP • Webinar to provide suggestions on ways of implementing SMS • Social media to provide information on SMS • Send patients reminders to use SMS via: ▪ E-mail ▪ Phone call (ex. when confirming next appointment)
Behavioural Regulation - Some patients don’t use SMS as much as they should	- Some patients did not use SMS as often as they should	• Encourage clinicians/interns to help patients form a SMART plan to self-manage • Encourage patients to put Post-it notes near their computer, automated recalls on their cell phone and/or lap-top • Send patients reminders to use SMS via: ▪ E-mail ▪ Phone call

#### Environmental context and resources

Ninety statements were associated with *Environmental Context and Resources* domain. These statements corresponded to 11 specific beliefs that were summarized under 5 themes: 1) time; 2) surrounding environment; 3) cost; 4) communication with clinicians; and 5) resources. Related to this domain, interviewees identified several barriers to using SMS including lack of time, lack of energy, work environment, and lack of educational materials. Interviewees also identified facilitators such as home environment, cooperation of clinicians, and receiving educational materials. All interviewees agreed that receiving educational/supportive materials on exercises would be helpful in adhering to SMS. However, about half of interviewees (7/13) said they had received educational materials from their treating interns. Most of the interviewees (10/13) indicated that they had difficulty performing exercises at work given the lack of space, and (6/13) stated that they would be able to do the exercises at home.

BCTs that were mapped to this domain included *environmental changes* and *time management*. For instance, providing patients with either paper-based or online handouts that describe and demonstrate relevant recommendations such as exercises or other life-style changes could help address this barrier. In addition, patients could be encouraged to seek advice from their intern/clinician on ways to manage their time so they may use SMS more efficiently. This could be offered by interns/clinicians during patient visits while using BAP, an intervention based on motivational interviewing.

#### Emotion

Thirty-one statements were mapped to the domain of *Emotion*. These statements represented 5 specific beliefs and were categorized under one theme: emotion concerning the use of SMS. More than half of interviewees (7/13) agreed that SMS made them feel good, though some interviewees stated that performing exercises, as a SMS component, sometimes exacerbated their pain and made them feel frustrated and anxious. Two patients felt anxious about not performing the exercises properly.

*Stress management* and *social support (emotional)* were identified as BCTs to address the barriers related to emotion. The intervention components proposed to implement these BCTs included: 1) providing the patients with educational/supportive materials (handout) on the use of SMS, and 2) encouraging patients to share successful strategies for reducing anxiety with other patients.

#### Memory, attention & decision making

Sixty-two statements pertained to the domain of *Memory, Attention & Decision Making*. These statements corresponded to 4 specific beliefs and 3 themes: ability to make a decision to use SMS, participating in decision-making on SMS, and remembering to use SMS. Almost half of interviewees (6/13) stated that remembering to use SMS regularly was challenging. Furthermore, almost all interviewees (12/13) preferred to be involved in their own clinical decision-making, as this may have motivated them to use SMS. They indicated that being involved in decision making would increase their adherence to SMS recommendations. Almost all interviewees (11/13) mentioned that they were very/somewhat involved in decisions about treatment options for SMS.

BCTs identified for this domain were *planning/implementation*; *prompts/triggers/cues*; and *motivational interviewing.* The suggested BAP technique could be led by the intern/clinician and used to deliver such BCTs to encourage patients to be more involved in the decision-making process. The BAP technique also provides patients with strategies to help them to remember to use and integrate SMS into their daily life.

#### Behavioural regulation

Forty-two statements were mapped to the *Behavioural Regulation* domain. These statements represented 3 specific beliefs forming one overarching theme: self-management is a part of patients’ routine. Most of the interviewees stated that they were integrating SMS into their routine. However, 8 out of 13 interviewees indicated that they did not use SMS as much as they could have because of lack of time, work commitments, feeling lazy, and forgetting to use SMS.

The BCTs and interventions mapped to this domain were similar to the ones mapped for the “*Memory, Attention & Decision Making*” domain. BAP technique emphasizing the SMART (Specific, Measurable, Attainable, Relevant, and Timely) goal setting approach can help patients create an action plan for routinely using SMS. In addition, reminder strategies were proposed to prompt patients to use SMS, including post-it notes placed near their computer, and automated recall messaging on their cell phone and/or lap-top. To facilitate self-monitoring, patients could also be invited to keep a journal or log their activity between visits.

#### Knowledge

Sixty-two statements were linked to the *Knowledge* domain. These statements represented 4 specific beliefs and collapsed into 2 themes: awareness of, and knowledge about SMS. All interviewees confirmed that they were aware of SMS and had sufficient information about it. However, interviewees tended to primarily focus on exercise, disregarding other components of SMS (e.g. lifestyle changes).

*Information regarding behaviour* was the BCT mapped to this domain. Patients identified the need to receive more information regarding SMS. The interventions proposed included distributing educational materials that summarized the key elements of SMS and emphasized ways to initiate/sustain lifestyle changes, as well as webinars and social media (e.g. Facebook) that would provide information on SMS.

#### Selection of the final knowledge translation (KT) intervention

Drawing upon current evidence for the effectiveness of different KT strategies [89] and BCTs [90] and the understanding of the feasibility of implementing interventions in the CMCC clinics, the research team members considered intervention components that would facilitate the use of SMS among patients with spine pain. They reached consensus on the following intervention components and modes of delivery:

I. Provide patients with supportive handouts summarising and demonstrating the therapeutic recommendations including exercises and other lifestyle changes.

- Paper-based
- Online: webinars or social media (e.g. Facebook)
- Videos

II. Create short videos for patients to share stories on their successful strategies and how they integrated SMS into their daily routine.
III. Implement a BAP program based on motivational interviewing led by interns/clinicians. This includes:

- SMART goal setting approach
- Advice on managing time
- Decision making process
- Reminder strategies

## Discussion

SMS programs aim to empower and prepare patients to take charge of their own health condition [91]. SMS can help decrease pain levels and improve physical function among patients with spine pain [17]. Our PAM survey results indicated that three-quarters of participants were already at a higher level of activation (Levels 3 & 4), while one quarter of participants were at a lower level of activation (Levels 1 & 2). Patients with high levels of activation need to be encouraged to continue adhering to SMS, whereas patients with low levels need help to increase their knowledge, motivation and self-efficacy in order to initiate behaviour change through the guidance of their healthcare provider. Highly activated patients are more likely to have positive health outcomes and lower health costs, and adopt new healthy behaviours [49]. In addition, a higher activation level is an indicator of patient adherence to SMS [49]. Level 3 suggests that although patients are changing their lifestyle, they still lack the confidence and skills to support such changes [49], while Level 4 indicates that the patients have already changed their lifestyle to support their condition, but this change may not be adequately maintained [49]. According to Hibbard and Gilburt (2014), the strategy needed for individuals in Levels 3 or 4 involves helping them adopt a new behaviour, and gain sufficient knowledge, confidence, and skills to be able to adopt and maintain the behaviour [49]. In addition, these patients need assistance to problem solve in difficult situations in order to maintain newly adopted behaviours [49]. On the other hand, people who have lower levels of PAM (i.e. levels 1 & 2) need assistance to understand their active role in the health care and increase their knowledge and confidence to participate in their health management [49]. These proposed strategies are consistent with the components of the interventions selected by our expert panel to address the identified barriers and help patients with chronic spine pain adhere to SMS.

In our study, we found that the adherence to SMS was influenced by barriers corresponding to 5 domains of the TDF, namely: *Environmental context and resources, Emotion, “Memory, Attention & Decision Making”, Behavioural Regulation,* and *Knowledge*. We identified two unique barriers that have not been previously identified, namely: *“Memory, Attention & Decision Making”* and *“Behavioural Regulation”*. In order to address the five aforementioned barriers, a panel of experts mapped BCTs to each barrier and selected the appropriate intervention components.

An additional barrier identified by interviewees was not having enough time to use SMS because of their various school, work and life commitments. This finding is consistent with May (2010) who showed that time and family commitments are external factors that restricted patients with spine pain from using SMS [45]. Half of interviewees expressed an interest in receiving supportive materials on SMS (describing the prescribed exercise), despite all of them confirming they had sufficient knowledge of SMS. However, interviewees focused only on exercise while disregarding other components of SMS (i.e. lifestyle changes). Poor access to information, not receiving effective information, or lack of support from clinicians regarding SMS are factors that have been reported to restrict the use of SMS among patients [45–47]. Therefore, providing patients with time management strategies and educational materials (e.g., paper based and e-pamphlets) summarizing the key components of SMS may provide further information on SMS and promote adherence to SMS [92, 93].

Poor mental health and low self-efficacy have been identified as internal factors restricting patient uptake of SMS [45]. Some interviewees indicated feeling anxious when performing therapeutic exercises, especially if they were unsure of how to perform them properly. Developing videos that demonstrate the prescribed home exercises may increase patients’ confidence to appropriately perform the exercises without supervision and reduce the likelihood of anxiety [94] toward SMS. In addition, having an online discussion board for patients may assist in reducing their anxiety through the sharing of helpful strategies and stories.

Although most interviewees confirmed they felt somewhat involved in the decision-making process, the majority indicated that they wanted to be much more involved in their treatment plan. Patients tend to value clinicians’ advice and recommendations [95], thus effective clinician-patient communication on health issues and setting treatment goals together may empower patients to adhere to their treatment plan, which may in turn increase their satisfaction with care and improve health outcomes (e.g. pain, functional ability, and emotional well-being) [96, 97]. Effective communication between patients and clinicians is an important component of SMS [98].

Lastly, interviewees indicated they felt confident and skilled to use SMS, believed in the benefits of SMS, intended to continue using SMS, and perceived SMS as an important approach to dealing with spine pain. This finding might be attributed to the fact that our study interviewees were already involved in, and may have benefited from, SMS. This could also explain why interviewees were optimistic about, and intended to use, SMS. These findings will facilitate the implementation of SMS among patients with spine pain in participating clinics as these patients are encouraged to be involved in SMS.

The expert panel suggested different KT intervention components based on BCTs to address the identified barriers to using SMS among spine pain patients. The selected KT intervention components formed a multifaceted theory-based program that could be implemented in addressing more than one barrier simultaneously [99]. Importantly, the KT intervention components (educational materials, webinar, videos that were developed by The CCGI (https://www.youtube.com/channel/UCduMXDBP76INn85Il9fQ3qg/playlists?view=50&shelf_id=2&sort=dd), BAP motivational interviewing, reminders, and use of social media) were selected based on the feasibility of their implementation within the respective clinical setting and evidence of their effectiveness. For example, a 2016 systematic review on patient-mediated knowledge translation interventions found educational materials can improve patients’ knowledge and support decision making [92]. Furthermore, Coudeyre et al. (2006) showed that using written materials with patients significantly improved their functional status and knowledge of spine pain [93]. Mahler (1999) reported that using videos as an educational tool improved treatment adherence to the exercise prescribed [100]. According to a logic model describing the effect of social media on behaviours, participation on social media may increase patient’s intention to adopt healthy behaviours, which in turn may lead to changes in behaviour and improvements in health outcomes [101]. Lastly, systematic reviews show that implementing multicomponent interventions (education, feedback, motivational intervening, counselling, and/or reminders) improves adherence to prescribed medications in different health conditions [102–104].

While CMCC promotes sustainable use of evidence-based practices (EBP) among graduates, a structured approach to SMS that allows for patient-centered goals is not yet integrated into the curriculum [105]. To promote the use of SMS at CMCC, the intervention targeting patients will be integrated with the theory-based KT intervention in order to enhance the use of SMS among clinicians and interns. The KT intervention designed in a parallel study by our expert panel (3 KT researchers, a researcher in medical education, 2 CMCC faculty members, and one patient representative) is composed of a webinar and online educational module on a SMS guided by the Brief Action Planning, clinical vignettes, training workshop, and opinion leader support [42]. We used the BAP framework to guide our SMS, which has been shown to enable its use [86, 87]. The use of SMS, guided by an action plan such as the BAP framework, has also been shown to improve patients’ adherence to recommended care [81] and health outcomes [98].

### Limitations

Our study has some limitations. We conducted the study in a large chiropractic teaching institution and as such, the results may not be generalized to all chiropractic private practice. Also, to keep interviews within a reasonable length, only 1 to 2 questions were used in the interviews to cover each of the TDF domains. As a consequence of this short interview format, the results may fail to capture the essence of each domain. Lastly, for the individual interview phase, only participants who responded to the invitation email were interviewed, these participants may not have been representative of the all study participants.

## Conclusions

Approximately a quarter of respondents seeking care for spine pain in chiropractic teaching clinics reported a lower level of activation in SMS. Theoretical barriers likely to influence the uptake of SMS among interviewed patients included: *Environmental Context and Resources; Memory, Attention & Decision-Making; Emotion; and Behavioural Regulation*. These findings informed the design of a multicomponent theory-based tailored KT intervention to optimize the quality of spine pain care and improve patients’ health outcomes.

## Additional files

Additional file 1: “Regression analysis and output”. It provides information about the regression analysis that was conducted to assess the influence of patients’ characteristics and patient activation. It also shows the output of the regression analysis. (DOCX 15 kb)

Additional file 2: “Barriers and facilitators to adherence of SMS among patients with back and neck pain - Interview topic guide”. It provides the interview guide for individual interviews with patients. (DOCX 19 kb)

Additional file 3: “PAM survey - raw patient data”. An Excel sheet provides the raw data for patients surveyed in phase 1 using the PAM instrument. (XLSX 53 kb)

Additional file 4: “Thematic analysis based on the TDF”. It provides number of patients’ statements for each TDF domain, TDF specific beliefs and themes. (DOCX 19 kb)

Additional file 5: “Specific Beliefs for each TDF with illustrative quotes”. It provides patient quotes representing specific TDF domains and beliefs. (DOCX 18 kb)

## Data Availability

The raw data that support the conclusions of this manuscript is available in the Additional file 3. The interview data are available in Additional files 4 and 5

## Abbreviation

BAP: Brief action planning
BCT: Behavioural change technique
CCGI: Canadian Chiropractic Guideline Initiative
CMCC: Canadian Memorial Chiropractic College
CPGs: Clinical practice guidelines
IQR: Interquartile range
KT: Knowledge translation
PAM: Patient activation measure
SMS: Self-management strategy
TDF: Theoretical domain framework
