# Supplementary material for: Promoting the use of self-management in patients with spine pain managed by chiropractors and chiropractic interns: barriers and design of a theory-based knowledge translation intervention
Source: Chiropr Man Therap. 2019 Oct 16;27:44. doi: 10.1186/s12998-019-0267-6 (PMC6794734; doi:10.1186/s12998-019-0267-6)
Supplement: Supplementary file 1 — “Regression analysis and output”. It provides information about the regression analysis that was conducted to assess the influence of patients’ characteristics and patient activation. It also shows the output of the regression analysis. (DOCX 15 kb) [file 12998_2019_267_MOESM1_ESM.docx]

Additional File1: “Regression analysis and output”

**Sample Size and** **Data Analysis**

Using simple and multiple linear regression analyses, we assessed factors that could affect the participation in SMS, including age, gender, duration of spine pain, previous chiropractic care, SMS knowledge, BAP knowledge, and presence of at least one comorbidity. Model coefficients (betas) were used to assess the association between PAM and other socio-demographic variables. All studied socio-demographic variables except age were treated as categorical variables.

Sample size needed to run a multiple regression analysis depends upon the number of predictor variables. The rule of thumb to calculate the sample size is (N ≥ 50+8m), where m refers to the number of number of coefficients in the regression model [1]. As this study included eight predictors, our sample size exceeded the 114 subjects needed to conduct the multiple regression [1, 2].

**Results**

The Multiple regression analyses suggested that having knowledge of SMS (B = 6.47, SE = 1.89, *P* = 0.0008) and knowledge of BAP (B = 8.18, SE = 3.78, *P* = 0.03) were significantly associated with a high score on the PAM. The table below presents the output of the multiple regression analysis.

| Variable | Estimate (B-Coefficient) | Standard Error (SE) | P-value |
| --- | --- | --- | --- |
| Age | - 0.04 | 0.07 | 0.56 |
| Gender Women (ref)  Men |  | | |
|  | 2.11 | 1.79 | 0.24 |
| Spine pain onset < 1 year (ref)  > one year |  | | |
|  | 1.61 | 2.21 | 0.47 |
| Receiving previous chiropractic care  Yes  No (ref) | 3.92 | 2.22 | 0.08 |
|  |  | | |
| Main health care provider:  Chiropractic intern  Licensed chiropractor (ref) | -1.73 | 4.14 | 0.68 |
|  |  | | |
| Previous knowledge on SMS: Yes  No (Ref) | 6.47 | 1.89 | 0.0008 |
|  |  | | |
| Previous knowledge on BAP: Yes  No (Ref) | 8.18 | 3.78 | 0.03 |
|  |  | | |
| Presence of other medical condition  Yes  No (Ref) | -1.46 | 1.94 | 0.45 |
|  |  | | |

Dependent Variable: PAM, Number of observation used = 206

**References**

1. Green, S.B., *How Many Subjects Does It Take To Do A Regression Analysis.* Multivariate Behav Res, 1991. **26**(3): p. 499-510.

2. Cohen, J., *Statistical power analysis for the behavioral sciences*. 1988, Hillsdale, N.J.: L. Erlbaum Associates.
