# Supplementary material for: Promoting the use of self-management in patients with spine pain managed by chiropractors and chiropractic interns: barriers and design of a theory-based knowledge translation intervention
Source: Chiropr Man Therap. 2019 Oct 16;27:44. doi: 10.1186/s12998-019-0267-6 (PMC6794734; doi:10.1186/s12998-019-0267-6)
Supplement: Supplementary file 2 — “Barriers and facilitators to adherence of SMS among patients with back and neck pain - Interview topic guide”. It provides the interview guide for individual interviews with patients. (DOCX 19 kb) [file 12998_2019_267_MOESM2_ESM.docx]

Additional File2: Barriers and facilitators to adherence of SMS among patients with back and neck pain - Interview topic guide

**A. Opening dialogue**

This interview will help us understand what helps or prevents you from managing your own pain. We are going to talk about things you may use to control the symptoms of your pain on your own. This can include taking frequent breaks, doing your home exercises, exercising regularly, taking care of your diet etc. The interview should take between 45-60 minutes. Do you have any questions at this point?

Throughout the interview I will be asking a series of 20 questions. At any time during the interview you can decide not to answer a question or you may choose to end the interview. To keep within the given timeframe I may stop you from further answering a question if I find that you have already addressed the question at hand.

Before we start, could you please tell me when were you diagnosed with back or neck pain? Do you still have it? How long did it last?

When we talk about interns in the interview, please remember this could also include the supervisory clinicians who are working with the interns at CMCC.

1. **Interview topic guide**

**Knowledge**

1. What do you understand about self-managing your back or neck pain? (Provide examples: exercise, keeping physically active). Do you feel you know enough?
2. What did you learn from your intern about self-managing your pain and how was this information given to you?

- Prompts: Examples of approaches are open discussions, pamphlets, PowerPoint presentations, videos, website, books, or a referral to a support group, etc.

**Skills**

1. What skills have you learned from your intern to help you self-manage your pain?
   - Prompt: Did they show you how to do exercises, or discuss how to have a healthy lifestyle?
2. Have you been able to apply this at home? If so, how?

- Prompt: What helps or prevents you from doing this?

**Beliefs about capabilities**

1. How confident are you in using the skills you have learned and the information you read about to self-manage your pain?
2. How comfortable are you in doing self-management at home?

**Beliefs about consequences and reinforcement**

1. What do you think are the benefits of self-managing your condition?

- Prompt: Does self-managing your pain help you to meet your personal goals?

1. What are the disadvantages of managing your pain yourself?

- Prompt: Does it hinder you in any way? Are there any negative consequences? Costs of missed work? Increased discomfort?

**Intention, goals and optimism**

1. What are your personal goals for your rehabilitation process?
2. Do you intend to continue self-management at home (on a scale of 1-10)? What would help to motivate you?
3. Do you feel optimistic about your condition?

**Memory, attention and decision processes**

1. How involved were you in deciding about your treatment options?

- Were you able to choose between different treatment options?

1. How do you feel about being involved in decision-making about your care? How does your involvement in decision-making affect whether or not you self-manage your pain? If you were to be more involved in decision-making about your care, do you think that would affect how much you self-manage your pain?

**Environmental context and resources**

1. Can you describe the physical aspects in your environment that help or prevent you from self-managing your pain (e.g., time, work, family, finances, etc.)?

- Prompt: Tell me about your home/work environment? Are those appropriate or not to self-managing your pain?

1. Did your intern provide you with any educational tools? (e.g. pamphlets, videos, apps)

- What characteristics did you like about those? (format, language)
- Which one was the most useful?
- Would you have any suggestions to improve education tools in general?

1. If you didn’t receive any educational tools from your intern, do you think it would have been beneficial to have some?

- What characteristics would you have liked?
- Do you have a preference for how you receive health information (e-mails, website links, twitter, Facebook, paper-based, other on tips on physical activity, gardening, nutrition, self-care) or its length (short vs. detailed)?

**Social influence**

1. How do the views of other people you know (e.g., family, friends, colleagues or other healthcare providers) or things you read about in the media influence your decision to self-manage your pain?

**Emotion**

1. How does self-managing your pain make you feel?

(e.g., encouraged, hopeful, discouraged, anxious)

1. Did you have the opportunity to discuss those feelings with your intern? If so, what was the result of that discussion?

**Behavioural regulation**

1. What steps would you say have you taken to integrate the idea of self-managing your pain into your life?

(e.g. What steps would you take to support your intention to take a walk if it started raining?)

1. Do you feel you use this as much as you should? Why?

This is the end of the interview. Are there any questions you want to reflect back on? Do you have any questions? Thank you for your time and participation.

1. **Demographic questions.**

Please answer the following questions:

- What is your date of birth (day, month, year)?
- What is your occupation and are you currently working?
