# Supplementary material for: Promoting the use of self-management in patients with spine pain managed by chiropractors and chiropractic interns: barriers and design of a theory-based knowledge translation intervention
Source: Chiropr Man Therap. 2019 Oct 16;27:44. doi: 10.1186/s12998-019-0267-6 (PMC6794734; doi:10.1186/s12998-019-0267-6)
Supplement: Supplementary file 4 — “Thematic analysis based on the TDF”. It provides number of patients’ statements for each TDF domain, TDF specific beliefs and themes. (DOCX 19 kb) [file 12998_2019_267_MOESM4_ESM.docx]

Additional File 4: Thematic analysis based on the TDF

| TDF domain | Questions (N) | Statements (N) | Specific beliefs (N) | Specific beliefs (number of utterances) | Increase* N (%) | Decrease* N (%) | No Influence* N (%) | Themes |
| --- | --- | --- | --- | --- | --- | --- | --- | --- |
| Knowledge | 2 | 62 | 4 | - I understand what using SMS for back or neck pain means **(N = 14)** - I know: enough **(N = 8)**, a fair amount **(N = 4)**, I don’t know enough **(N = 1)** about SMS - I was taught about SMS techniques for exercises, posture, and/or diet **(N = 24)** - I was **(N = 9)** / I wasn’t **(N = 2)** provided with handouts on SMS techniques | 59 (95.2%) | 3 (4.8%) | - | Awareness of SM, knowledge about SM |
| Skills | 2 | 19 | 1 | - I have the skills to: do the exercises **(N = 16),** take care of my diet **(N = 3)** | 19 (100%) | - | - | Having skills to use SM |
| Beliefs about Capabilities | 2 | 33 | 3 | - I am very confident / quite confident / confident in managing spine pain using SMS **(N = 10)**, fairly confident **(N = 5)** - I am very comfortable / quite comfortable / comfortable in managing spine pain using SMS **(N = 12)/** fairly comfortable **( N = 1)** - I am able to do exercises **(N = 2)**, it is difficult to do some exercises **(N = 3)** | 30 (90.9%) | 3 (9.1%) | - | Acceptance, capabilities |
| Optimism | 1 | 15 | 1 | - I’m optimistic **(N = 9)**, fairly optimistic **(N = 4)**, not optimistic **(N = 2)** about my condition | 13 (86.7%) | 2 (13.3%) | - | Optimism about back pain |
| Beliefs about Consequences | 2 | 36 | 4 | - Benefits of SMS include **(N = 24)**: reduced pain, greater function, less stress and worry, meeting personal goals, greater awareness of things that cause pain, independency, fewer visits to HCP and reduced cost, saving time, reduced tiredness, prevention of injury, greater work productivity, more autonomy and control over my health, amplifying and sustaining the effect of treatment - There are no disadvantages to using SMS **(N = 7)** - SMS hasn't had any impact **(N = 1)** - Exercise can aggravate the pain **( N = 3),** SMS can be isolating **(N = 1)** | 31 (86.1%) | 4 (11.1%) | 1 (2.8%) | Consequence of managing spine pain patients with SMS |
| Reinforcement | 1 | 17 | 1 | - Factors reinforcing the use of SMS include: choosing to live a healthy lifestyle and feeling good **(N = 4),** being pain-free **(N = 3)**, doing my exercises with the intern **(N = 2),** having independent life style **(N = 4)**, having exercise videos (guide) **(N = 1),** being in a good shape **(N = 1),** knowing the benefits of SMS **(N = 2)** | 17 (100%) | - | - | Reinforcement of using SMS |
| Intention | 1 | 14 | 1 | - I intend or strongly intend **(N = 13)**, I do not intend **(N = 1)** to continue with SMS | 14 (100%) | - | - | Intention to use SMS |
| Goals | 1 | 17 | 3 | - SMS is important/ a priority to me **(N = 4)** - My goals are to manage pain **(N = 11)**, to be able to do the activities **(N = 1)**, to be more flexible **(N = 1)** | 17 (100%) | - | - | Patients’ goals |
| Memory, attention & decision making | 2 | 62 | 4 | - I was very/somewhat involved in decisions about treatment options for SMS **(N = 12),** I wasn’t involved **(N = 2)**, I do not need to be more involved in decision-making **(N = 1)** - I was **(N = 13)** / I was not able **(N = 3)** to choose between treatment options, I was not provided with treatment options **(N = 2)** - Being involved in decision-making is: important to adhere to SMS **(N = 14)**, motivated/empowered me **(N = 6)**, is helpful **(N = 1)**, increases my confidence in using SMS **(N = 1)** - Remembering to do SMS is challenging **(N = 6)**; setting reminders somewhat helps me to apply SMS at home **(N = 1)** | 48 (77.4%) | 14 (22.6%) | - | decision to use SMS, participating in decision-making on SMS |
| Environmental context and resources | 2 | 90 | 11 | - I lack time to use SMS **(N = 19)** - Having time **(N = 2)** or scheduling SMS facilitates its use **(N = 1)** - I lack of energy to use SMS **(N = 5)** - Work environment restricts **(N = 10)** / facilitates **(N = 1)** the use of SMS - Rain restricts the use of SMS **(N = 1)** - I am able to use SMS at home **(N = 6)**, nothing stops me from using SMS **(N = 1)** - Having a gym close to me facilitates the use of SMS **(N = 2)** - Financial cost restricts the use of SMS **(N = 4)** - Difficulty to contact the intern/chiro between visits restricts the use of SMS (N = 1) - The health care team is collaborative **(N = 4)**, having someone to guide me facilitates the use of SMS **(N = 1)** - Being with a group motivates me to do exercises **(N = 2)** - I did not receive **(N = 5) /** I received educational materials **(N = 7)** - Receiving educational material is helpful **(N = 15)** - Reminders would help me use SMS **(N = 2)** | 45 (50%) | 45 (50%) | - | time, surrounding environment, cost, communication with clinicians, availability of resources |
| Social Influence | 1 | 19 | 3 | - My family supports me using SMS **(N = 3)** / although some family members don't believe I need SMS, that doesn't influence me to use it **(N = 1)** - I seek help from healthcare providers and people on using SMS **(N = 3)** - Media and friends encourage me to use SMS **(N = 8) /** media doesn’t influence me **(N = 3) /** things I read or hear in the media or from friends helped me in decision making **(N = 1)** | 15 (78.9%) | 1 (5.3%) | 3 (15.8%) | Influence of others (family, friends, HCP, media) |
| Emotion | 2 | 31 | 5 | - Using SMS makes me feel: empowered **(N = 3),** good **(N = 7) /** anxious **(N = 2)** / not anxious **(N = 1)**, can be frustrating **(N = 4),** optimistic/hopeful/positive **(N = 5)** - My intern encourages me to remain positive **(N = 2)** - I discussed **(N = 3) /** I haven’t discussed my feelings with my intern **(N = 4)** | 21 (67.7%) | 10 (32.3%) | - | Emotion toward use SMS |
| Behavioural Regulation | 2 | 42 | 3 | - I am integrating SMS into my routine **(N = 27)** - I use SMS as much as I can **(N = 5) /** I don't use SMS as much as I should **(N = 8)** - I discussed my progress with my intern **(N = 2)** | 34 (81%) | 8 (19%) | - | SMS is a part of life routine |

* increase (facilitator), decrease (barrier), or no influence on the use of SMS. HCP: healthcare provider, SMS: self-management support, TDF: theoretical domain framework.
