# Supplementary material for: Promoting the use of self-management in patients with spine pain managed by chiropractors and chiropractic interns: barriers and design of a theory-based knowledge translation intervention
Source: Chiropr Man Therap. 2019 Oct 16;27:44. doi: 10.1186/s12998-019-0267-6 (PMC6794734; doi:10.1186/s12998-019-0267-6)
Supplement: Supplementary file 5 — “Specific Beliefs for each TDF with illustrative quotes”. It provides patient quotes representing specific TDF domains and beliefs. (DOCX 18 kb) [file 12998_2019_267_MOESM5_ESM.docx]

Additional File 5: Specific Beliefs for each TDF with illustrative quotes

| TDF domain | Specific beliefs (number of utterances) | Selected Statements by participants |
| --- | --- | --- |
| Knowledge | Patients understand what is self-management for spine pain | *“It means to be engaged with a care plan. It means to keep myself motivated, to maintain structures that are supportive of pain management for example. Exercising, making sure that I take my anti-inflammatories and doing things like meditation which I’m not very rigorous about. It’s about engaging in one’s own care.”* |
|  | Patients know enough about SM | *“I personally do (know enough). … I’m always open to learning more but I think I do a fairly good job”* |
|  | I was taught SMS techniques such as exercises, posture, and/or diet | *“They’ve taught me how to sit better so I don’t slouch and cause my neck pain to get worse. They’ve taught me some exercises to do to improve my core. They stressed the importance of eating healthy…”* |
|  | I was provided with handouts on SMS techniques | *“I’ve been given paperwork where I can read a little more about it as well as forcing me to demonstrate my understanding of a stretch or the exercise”* |
| Skills | I have the skills needed to do the exercises, take care of my diet | *“One of the things I’ve learned is... if I’m in pain I could say elevation if it’s in the leg; neck exercises I could say pulling your chin together, tucking it in. Different posture techniques. Proper squatting”* |
| Beliefs about Capabilities | I am very confident / quite confident/ confident in managing spine pain using SMS | *“I’m very confident. I think I know what I need to do and usually I do it”* |
|  | I am very comfortable/ quite comfortable/ comfortable in managing spine pain using SMS | *“I am very comfortable with it is because I was doing lots of it before”* |
|  | I am able to do the exercises/ it is difficult to do some exercises | *“Oh yes, yes, I do the exercises regularly and of course the heel lift I’ve been using. It got easier, the exercises got easier as I stretched more, yeah.”*  *“Some exercises are difficult to do on your own at home as opposed to having someone execute them on you at treatment”* |
| Optimism | I am optimistic / not optimistic about my condition | *“Lately I feel very optimistic”* |
| Beliefs about Consequences | Benefits of SMS | *“Well hopefully, the best bit is not having to go to the doctor, and that ends up being a waste of time, and sometimes that results in being prescribed medication that you can avoid taking, so I think the benefit is when I feel (inaudible) I know to do these movements to relieve the discomfort and so the benefits are immediate. It’s just to stay healthy and be pain-free”* |
|  | There are no disadvantages to using SMS | *“I don’t think so,…* [there are] *no disadvantages”* |
|  | SMS hasn't had any impact | *“Has it had any impact on things that you can do that maybe you couldn’t do before? No I don’t think so”* |
|  | Exercise can aggravate the pain**,** SMS can be isolating | *“I may do some exercise that may aggravate the pain”* |
| Reinforcement | Factors reinforce the use of SMS | *“Well nobody else is there to look after me. So I have to be a self-manager”* |
| Intention | I intend/ I strongly intend to continue with SM | *“Yes* [I intend to continue self-management at home], *because my health comes first. I don’t want to suffer in the long run. I think 8-9”* [on a scale of 1-10] |
| Goals | SMS is important to me | *“I understand that it’s important for my daily life and the activities that I want to do”* |
|  | SMS is a priority | *“Self-care and self-management of these things that I know will be persisting because I know that they’re related to my lifestyle and my job. I’ve learned to start prioritizing these things so that I don’t get to a state where it’s causing me to change the way I have to live my life”* |
|  | Patients’ goals are to manage pain, to be able to do the activities, or to be more flexible | *“My primary goal is to try to avoid any kind of reliance on opioid pharmaceuticals or drugs to manage pain. I don’t take any at this point and I don’t want to”* |
| Memory, attention & decision making | I was very / somewhat involved in decisions about treatment options for SMS | *“I was very involved. I listened to their suggestions and I was very vocal about what I wanted to do and not want to do”* |
|  | I was able to choose between treatment options | *“Yes, they wanted me to have adjustments done, and there was this other tool they wanted to use and I said no, I’d rather not, even though a lot of people have adjustments I just don’t want to. It’s more comfortable for me so, I did have a say in it. Because they were the professionals and they know, I didn’t know exactly what to get”* |
|  | Being involved in decision-making is important to adhere to SMS / motivated me / empowered me / was helpful / it increases my confidence in using SM | *“I think it’s extremely important. I don’t think a doctor should tell you. This is what’s right for you’ without giving you any choice in the matter. I want to know the options and I want to decide for myself what’s right for me”* |
|  | Remembering to do SMS is challenging | *“But it’s challenging to actually remember that I’m actually supposed to do it every week. I typically remember once I’m lying in bed and it’s too late”* |
| Environmental context and resources | I lack time to use SMS | *“Yes, all of the above. I am in school so being a student I have to make a conscious effort to take time out of my day or even my week to find the time to go in. That does prove difficult”* |
|  | I lack energy to use SMS | *“For me it’s mostly in my shift work. I’m a nurse. I do shift work. So sometimes that prevents me from exercising. Because I’m too tired or I get called into work and I wasn’t supposed to be working. That might prevent me from working out as regularly as I would like to”* |
|  | Work environment restricts the use of SMS**,** I am able to use SMS at home | *“If I’m sitting in an office, even though one of the days I’m on my own, I can’t start lying on the floor (to do the) exercises”* |
|  | Financial cost restricts the use of SMS | *“Yes, finances, yes. Finances is an issue also.”* |
|  | The health care team is collaborative | *“I just want to say that all of the interns that I’ve seen and all of the staff that I’ve seen at the office have been extremely nice”* |
|  | I did not receive / I received educational materials | *“No, I haven’t received that from my CMCC intern, I have received lots of that kind of pamphlets, workbooks and website to review with my cardiac rehab program”* |
|  | Receiving educational material would be helpful | *“As far as my chiropractic care right now, I think I would appreciate maybe more educational material or educational information to help me along with the physical exercise I’m doing, I think that would maybe motivate me to do more or keep it up”* |
| Social Influence | My family supports me in using SMS**.** Although some of my family members don't believe there is a need for SMS, that doesn't influence me | *“Both my family doesn’t have a lot of time to take care of themselves and their pain and they don’t do it but I think they look up to me and they are very supportive of what I do and my decisions I suppose”* |
|  | I seek help from HCP and people on using SMS | *“So when I came there I was telling I can’t remember which one of the doctors I had at the time, and they said “No, it’s too hard.” So they advised me to buy a memory foam mattress topper, which I did, and I sleep like a baby now. So that’s one of the examples where people say, “Oh, do this,” and I did it, and I ended up having to go back to the healthcare provider for advice”* |
|  | Media and friends encourage me to use SM**,** media doesn’t influence me | *“I think there is a lot of media that supports the idea of self-care and taking time for yourself and I think there’s a lot of resources online for exercises and things to self-treat and self-diagnose injuries”* |
| Emotion | SMS makes me feel empowered | *“Good. I like to be proactive about things in my life, I want to be in control of my life. So managing my health and my body is part of that”* |
|  | SMS makes me feel good | *“Makes me feel better about myself, that I’m actually taking care of myself better”* |
|  | SMS makes me feel anxious / not anxious | *“You feel anxious that you’re maybe not doing the exercises right? Yeah it’s like why is this still painful, why am I doing this exercise, I’m still painful. I become very impatient and I like to have the support of someone like a massage therapist or a chiropractor or my intern.”*  *“Very good. Hopeful. Very hopeful. (So you don’t feel anxious about it?) No, not at all.”* |
|  | SM can be frustrating**,** makes me optimistic/hopeful/positive. My intern encourages me to remain positive | *(You’ve talked about not being able to do it sometimes at work, does that make you feel frustrated?) “Well I’ve just got to live with it, I’m not going to be able to change it”*  *“I feel optimistic.”* |
|  | I discussed / I haven’t discussed my feelings with my intern | *“Yes. We always have discussions and I talk to my interns about what I’ve learned and they look into it further”* |
| Behavioural Regulation | I am integrating SMS into my routine | *“Yeah, so from morning I do the stretches that – because the night is the worst, so in the morning I’m, you know like at night-time if I stay in the same position sleeping for a long time that, I’m just worse in the morning, so that’s why I do those stretches and then right away I feel better”* |
|  | I use SMS as much as I can**,** I don't use SMS as much as I should | *“No. I feel like I could be better and more diligent about doing exercises regularly. I would say probably about 55% of the time I feel like I’m doing a good job”*  *“I don’t think I should do more, that’s actually been a challenge for me. This is just specific for me but I think I’m prone to”* |
|  | I discussed my progress with my intern | *“Another thing I have done, if I’m not sure or between visits if I have a concern, I try to speak to my intern over the phone. It’s not always easy. Another thing is I wish there was emailing too, like with pain management, if there’s something you could communicate via email to your intern”* |

CMCC: Canadian Memorial Chiropractic College, HCP: healthcare provider, SMS: self-management support, TDF: theoretical domain framework.
